# Supplementary material for: Preparation, Characterization and Multiple Biological Properties of Peptide-Modified Cerium Oxide Nanoparticles
Source: Biomolecules. 2022 Sep 10;12(9):1277. doi: 10.3390/biom12091277 (PMC9496055; doi:10.3390/biom12091277)
Supplement: Supplementary file 1 [file biomolecules-12-01277-s001.zip › biomolecules-1904647-supplementary.pdf]

Supplementary Material

Preparation, Characterization and Multiple Biological Properties of Peptide-Modified Cerium Oxide Nanoparticles

Mengjun Wang †, Hongliang He †, Di Liu, Ming Ma * and Yu Zhang *

State Key Laboratory of Bioelectronics, Jiangsu Key Laboratory for Biomaterials and Devices, School of Biological Sciences and Medical Engineering, Southeast University, Nanjing 210096, China

* Correspondence: zhangyu@seu.edu.cn (Y.Z.); maming@seu.edu.cn (M.M.)
† These authors contributed equally to this work.

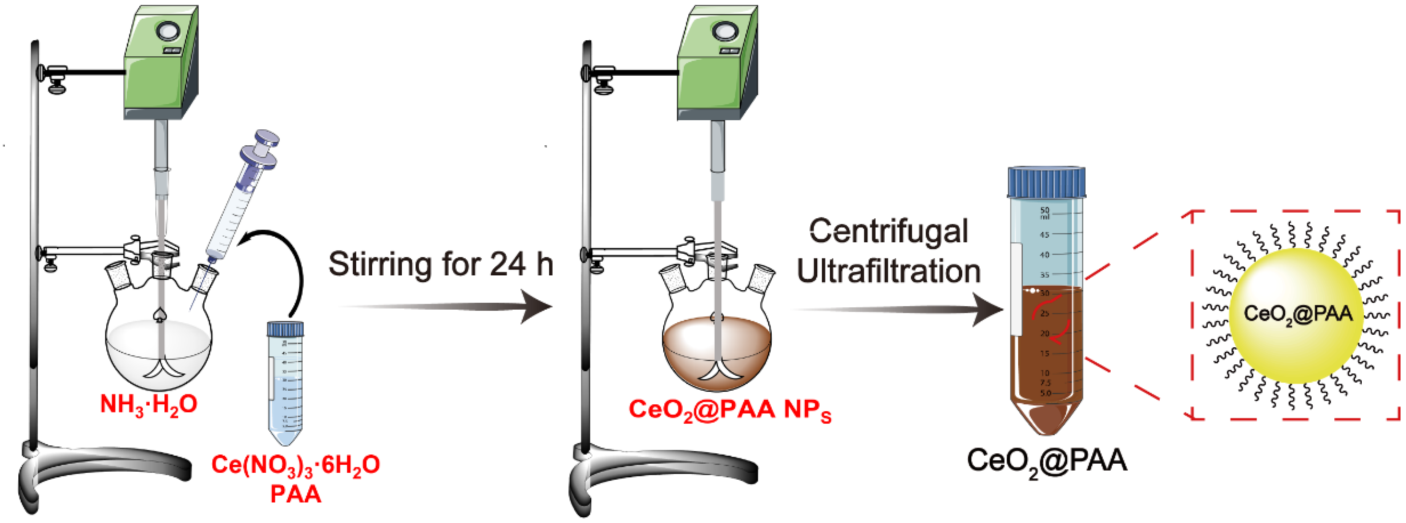

Figure S1. Schematic illustration of the preparation of CeO_2 @PAA NPs.

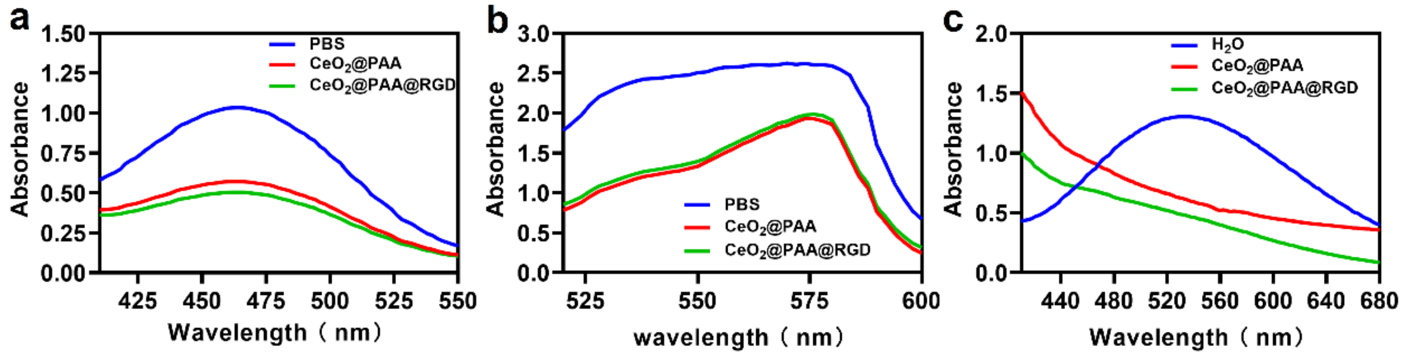

Figure S2. UV-vis absorption spectra of CeO_2@PAA and CeO_2@PAA@RGD towards (a) $O_2^{\cdot-}$ and (b) H_2O_2 and (c) ·OH.

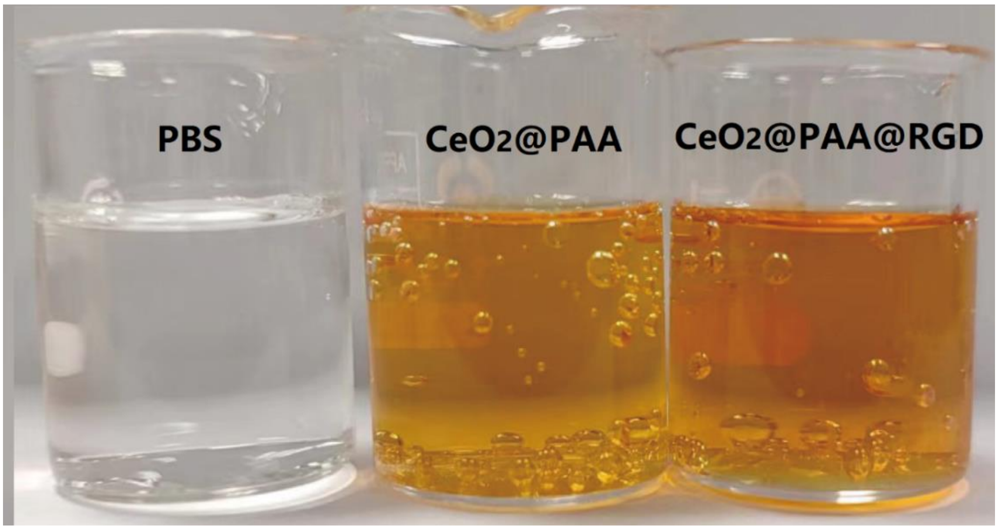

Figure S3. *In vitro* oxygen bubbles production with a significant yellow colour in nanoparticles to assess CAT-like activity.

Table S1. Reaction system for the detection of SOD-like enzyme activity

Reagents	Sample	Blank control 1	Blank control 2	Blank control 3
Sample (μL)	2	-	-	2
SOD assay buffer (μL)	-	2	22	20
WST-8/enzyme working solution (μL)	160	160	160	160
Reaction start working fluid (μL)	20	20	-	-

Table S2. Reaction systems for the detection of ·OH

Reagents	A_0 (μL)	A_x (μL)	A_{x0} (μL)
$FeSO_4$	50	50	50
Ethanol-salicylic acid	50	50	50
Distilled water	15	-	50
Sample (10 mg/mL)	-	15	15
H_2O_2 (30%)	50	50	-

Table S3. Reaction system for the synthesis of cDNA by reverse transcription.

Component	Volume
5 x Reaction Buffer	4 μL
Oligo $(dT)_{18}$ Primer (100 μM)	0.5 μL
And Random Hexamer primer (100 μM)	0.5 μL
Servicebio®RT Enzyme Mix	1 μL
Total RNA *	10 μL
RNase free water	Add to 20 μL

Table S4. Primers used to detect mRNA expression of pro-inflammatory and anti-inflammatory related genes.

Gene	Forward Primers	Revers Primers
CD86	CTGGACTCTACGACTTCACAATG	AGTTGGCGATCACTGAGAGTT
TNF-α	CCTGTAGCCCACGTCGTAGC	AGCAATGACTCCAAAGTAGACC
IL-6	ATCCAGTTGCCTTCTTGGGACTGA	TTGGATGGTCTTGGTCCTTAGCCA
CD206	CTGCAGATGGGTGGGTTATT	GGCATTGATGCTGCTGTTATG
VEGF	AGGAGAACTGCTGTGTACGC	CCGTGCTATGGGTTATGTCT
IL-10	ACTGGCATGAGGATCAGCAG	CTCCTTGATTTCTGGGCCAT
